# Modeling the dynamics of oligodendrocyte precursor cells and the genesis of gliomas

**DOI:** 10.1371/journal.pcbi.1005977

**Published:** 2018-03-28

**Authors:** Aloys Dufour, Emilie Gontran, Christophe Deroulers, Pascale Varlet, Johan Pallud, Basile Grammaticos, Mathilde Badoual

**Affiliations:** 1 IMNC Laboratory, CNRS, Univ Paris Saclay, Univ Paris-Sud, Univ Paris Diderot, France; 2 Department of Neuropathology, Sainte-Anne Hospital, IMA-Brain, INSERM U894, Univ Paris Descartes, Paris, France; 3 Department of Neurosurgery, Sainte-Anne Hospital, IMA-Brain, INSERM U894, Univ Paris Descartes, Paris, France; Oxford, UNITED KINGDOM

## Abstract

Oligodendrocyte precursor cells (OPCs) have remarkable properties: they represent the most abundant cycling cell population in the adult normal brain and they manage to achieve a uniform and constant density throughout the adult brain. This equilibrium is obtained by the interplay of four processes: division, differentiation or death, migration and active self-repulsion. They are also strongly suspected to be at the origin of gliomas, when their equilibrium is disrupted. In this article, we present a model of the dynamics of OPCs, first in a normal tissue. This model is based on a cellular automaton and its rules are mimicking the ones that regulate the dynamics of real OPCs. The model is able to reproduce the homeostasis of the cell population, with the maintenance of a constant and uniform cell density and the healing of a lesion. We show that there exists a fair quantitative agreement between the simulated and experimental parameters, such as the cell velocity, the time taken to close a lesion, and the duration of the cell cycle. We present three possible scenarios of disruption of the equilibrium: the appearance of an over-proliferating cell, of a deadless/non-differentiating cell, or of a cell that lost any contact-inhibition. We show that the appearance of an over-proliferating cell is sufficient to trigger the growth of a tumor that has low-grade glioma features: an invasive behaviour, a linear radial growth of the tumor with a corresponding growth velocity of less than 2 mm per year, as well a cell density at the center which exceeds the one in normal tissue by a factor of less than two. The loss of contact inhibition leads to a more high-grade-like glioma. The results of our model contribute to the body of evidence that identify OPCs as possible cells of origin of gliomas.

## Author summary

Gliomas are the most common brain tumors and result in more years of life lost than any other tumor. Standard treatments only confer a limited improvement in overall survival, underscoring the need for new therapies. Finding the type of cells at the origin of these tumors could lead to the development of new drugs, specifically targeted towards these cells. The oligodendrocyte precursor cells are suspected to be these cells of origin, because they continue to proliferate through all the adult life. In this article, we present a model of the dynamics of these cells, first in the normal brain, and then we extrapolate our model to the pathological situation. We study several scenarios where, from the normal situation, a cell appears with one property different from those of the normal cells. We show that the alteration of only one of the properties of these cells in the model can lead to the formation of gliomas with different aggressiveness and very similar to real gliomas, reinforcing the suspicion that the precursor cells are at the origin of gliomas.

## Introduction

Contrary to a long-lasting belief, there exists a population of proliferating cells everywhere in the mammalian adult brain [1, 2]. These cells are the oligodendrocyte precursor cells (OPCs) and they differentiate into oligodendrocytes [3], but also in some circumstances to astrocytes. They can be found everywhere in the central nervous system, in the gray and white matter [1], contrary to neurogenic areas that are mostly situated in the hippocampus, the olfactory bulb and all along the lateral ventricule [4]. OPCs represent the most important cycling population in the adult human normal brain [1]. In the mouse, recent studies have revealed that their density is strikingly uniform throughout the brain and that this is achieved through self-repulsion mediated by contact-inhibition [5]. OPCs constantly survey their surroundings by actively extending and retracting filopodia. Growing filopodia retract when they contact another filopodium, causing the cells to be equally spaced from one another and to occupy non-overlapping domains. Their density is also remarkably constant in time: the cells achieve the homeostasis of the normal brain tissue by balancing the differentiation and death process by proliferation, which allows them to keep their density constant on average.

Through this dynamical interplay between proliferation, differentiation and death, the precursors can be very rapidly mobilised after a brain injury that has killed mature oligodendrocytes. The surrounding OPCs proliferate and migrate to the lesion area, where they differentiate and replenish the injured area by new mature oligodendrocytes. This is indeed what happens in the case of an acute (such as in traumatic or vascular accidents) demyelinisation episode. The case of multiple sclerosis is more complex, since an active remyelinisation can be performed in early lesions, but is completely inhibited in chronic ones. This disease is thus characterized by the existence of large demyelinated areas that do not heal, leading to serious disability for the patients [6].

The oligodendrocyte precursor cells are suspected to be at the origin of some gliomas. Gliomas account for almost 80% of primary malignant brain tumors, and they result in more years of life lost than any other tumor [7]. Standard treatment only confers a modest improvement in progression and overall survival [8], underscoring the pressing need for the development of new therapies. Gliomas are classified with respect to their malignancy from grade I to IV by the World Health Organization [9]. From grade II, gliomas are invasive, and isolated glioma cells can be found beyond the MRI limits of signal abnormalities, including T2-weighted and FLAIR sequences [10, 11]. This characteristic explains the systematic recurrences that are observed after oncological treatments. Diffuse low-grade gliomas, i.e. grade II gliomas, are primary brain tumours that affect young adults, contrary to high-grade glioma that is more frequent in elderly people. At the beginning of their evolution, the low-grade gliomas grow slowly and continuously without angiogenesis [12]. During this phase, we have shown that the cell density in the tumor center is higher than in the normal tissue, but stays moderate. The number of proliferating cells in the tissue also remains low [11]. In [13] a diffusion-proliferation model has been used to estimate the date of birth of low-grade gliomas, and a side result was an estimate of the proliferation coefficient around 1 yr^−1^. During this slow-growing phase, the mean radius of these tumours measured on MR images increases linearly with time [14, 15]. After several years of growth, and despite treatments (surgery, chemotherapy and radiotherapy), angiogenesis is suddenly triggered and low-grade gliomas inexorably evolve into more aggressive forms (glioblastoma multiforme), impeding the social and professional life of the patients [16]. Clearly, new therapeutic strategies are urgently needed. Identifying the cell of origin of gliomas (that could be specific to each type of glioma) could constitute one of these new approaches, by inducing the development of targeted drugs against a specific type of cells. Targeted drugs could be expected to be more efficient against the tumor cells, but to have less side effects on normal tissue than non-specific drugs.

Many gliomas express markers characteristic of oligodendrocyte progenitors and the fact that OPCs remain widespread in the adult brain and still actively proliferate in the adult central nervous system make them good candidates for the accumulation of mutations and thus gliomagenesis [17–19]. The high ability of these progenitors to regulate their proliferation according to exogeneous signals may play a role in their susceptibility to transformation. Indeed, in vitro, it is possible to break the homeostasis by adding a growth factor (PDGF) and trigger uncontrolled proliferation: in vivo, in rodents, overexpression of PDGF is often used to induce gliomas [20]. This hypothesis is supported by several experiments with murine animals that show that the OPCs can become highly proliferative and form a malignant tumour after reactivation by inducing mutations [18, 21, 22].

In the domain of cancer research, mathematical models have been developed for decades to help address the essential questions on tumor growth (for a review, see [23]). At the genetic scale, in the pre-cancerous step, some models describe the dynamics of mutation accumulation that lead to cancer [24, 25]. At the cellular scale, other models describe how mutations (or phenotypic changes) can disrupt the homeostasis of hierarchically organized tissues [26–29]. At the tumor level, yet other models focus on the tumor growth dynamics after the appearance of the first tumor cells, their interaction with the micro-environment and the influence of treatments, with discrete [30, 31], continuous [32–34] and hybrid approaches [35–37].

The model we introduce here belongs to the class of models at the cellular level, and it addresses the problem of the cell-of-origin. We focus on a very specific tumor (gliomas) and a specific type of precursor cells (OPCs). The stem cells and progenitors density in hierarchically organized tissues such as hematopoietic system, epithelium or colonic crypts is regulated by a more differentiated downstream cell population [38], but this is not the case for OPCs that regulate their density by themselves, through contact inhibition and a density-dependent lifetime. To our knowledge, there is no mathematical model of the dynamics of normal OPCs and the disruption of their homeostasis that leads to gliomas. We therefore designed a specific and original model of the dynamics of these progenitor cells, based on a cellular automaton including the main processes that the OPCs undergo, i.e. self-repulsion, proliferation, migration, differentiation/death. We show that our model is able to reproduce the homeostasis and the repair process that characterize OPCs. In a second part of the paper, we describe the possible genesis of a glioma, by modeling the appearance of an over-proliferating cell, a non-proliferating cell or a cell that has lost its contact inhibition. When a tumor develops, we compare its characteristics to histological samples of human low-grade gliomas.

## Material and methods

### The model

**Fig 1:**
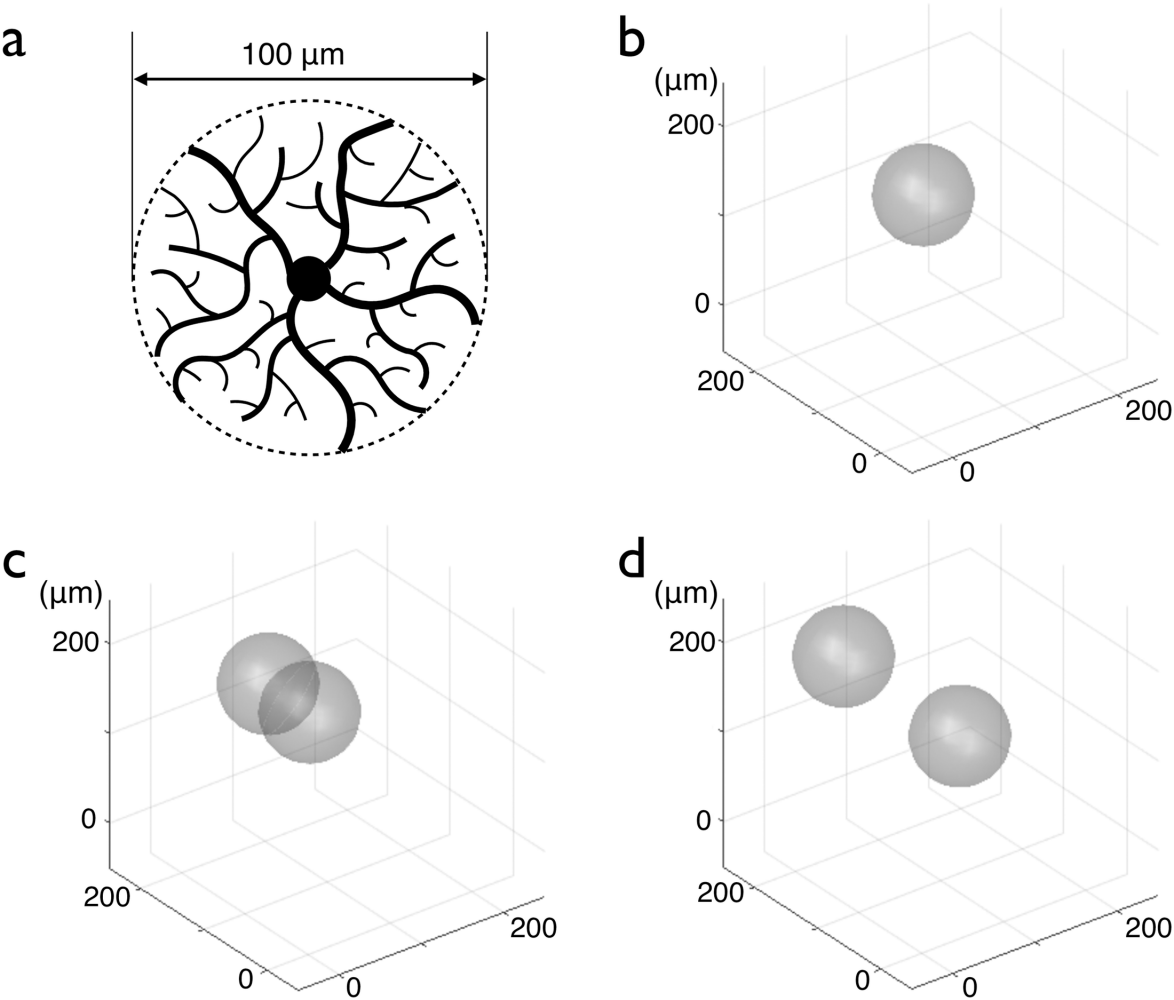
Model of an OPC and illustration of the proliferation/migration rules. (a) Schematic drawing of an OPC where the cell center and the filipodia are visible. The cell is modeled by a 100 *μ*m diameter sphere, represented by a dashed line drawn around the cell extensions. (b) A cell without overlap with other cells keeps moving with a constant velocity in the same direction. The direction of the motion changes only when the cell has overlaps with other cells. (c) The cell undergoes mitosis: a new cell is created and its center is placed at the distance *R* from the center of the first cell. (d) The two cells move in opposite direction in order to reduce the overlapping. After separation, they keep moving in the same direction, at a constant velocity.

Our automaton model consists in a collection of cells which evolve according to a given set of rules. Each cell is modelled by a sphere. The center of the sphere represents the cell soma (modelled as a point), and the sphere models all the filopodia and extensions around it, see Fig 1(a). The cell radius is the same for all cells in a given simulation and its value, typically *R* = 50 *μ*m, stays constant during the simulation. The three-dimensional space is continuous, i.e. the position of a cell in space is a triplet of real numbers, the coordinates of the center of the sphere. In order to avoid border issues, we choose periodic boundary conditions. At each iteration, the cells are updated, one after the other, in a random order in order to avoid undesirable correlations. At each iteration, each cell can participate in three different processes: proliferation, migration, differentiation/death (described in what follows). To be able to track individual cells easily, a number is attributed to each cell and stays the same for the whole life of the cell.

#### Proliferation

First, the cell has to choose, with a probability 0.5, whether it enters the proliferation process or if it moves. We choose to model the proliferation process as a poissonian process. If it enters the proliferation process, the cell has a probability 2λ to proliferate at each iteration. At each iteration, for each cell, a random number *r* is picked. If *r* < 2λ, then the cell divides and a new cell is created; otherwise, the cell does not divide. If the cell divides, a random direction is chosen. The position of the point given by this direction, at a distance *R* of the center of the cell, gives the position of the center of the newly created cell. If the distances between the center of this new cell and each of the cells that overlap the first one are all larger than 2*R*, only then the new cell is indeed created. This condition is necessary so that the newly created cell is not placed over other cells. This rule models the saturation of proliferation at high density: if the cells are already overlapping, they should not be able to proliferate more. This rule is in agreement with the experimental evidence that oligodendrocyte precursors closely regulate their numbers through interactions between adjacent precursors [39].

The proliferation of a cell is represented in Fig 1: in (b), the cell is alone. Then, in (c), a new cell is created with its center at a distance *R* of the center of the first cell. There is overlapping between the two cells so they move away from each other (d). This process of migration is explained in the next paragraph.

#### Migration

If the cell chooses to enter the migration process, it will move over a constant distance (typically a few percent of *R*) per iteration, in a direction conditioned by the different overlapping situations the cell experiences. To model the repulsion between two cells by contact inhibition, we introduce a migration-repulsion rule, so that a cell the extensions of which overlap with the extensions of another cell, will move away, in order to reduce these overlaps. If extensions of cell 1 overlap the extensions of cell 2 (i.e. if the distance between the positions of the two cells is smaller than 2*R*), cell 1 will move along the line defined by the position of the two cell centers, away from the other cell, at constant velocity. In Fig 1(d), after the proliferation and the separation of the two cells, they still move in the direction they had before the separation. The fact that cells keep moving when they are isolated is a way to reproduce migrating behaviours seen experimentally, for instance when cells migrate to replenish a lesion or during development [5, 40].

#### Differentiation/Death

In [5], either death or differentiation is characterised by the decrease (progressive in the case of differentiation, abrupt in the case of death) of the expression of NG2, the shrinkage ending with the disappearance of the cell domain. We thus model the two phenomena together with a unique rule. This process is modelled by implementing a lifetime clock [41]. The simplest way to conceive this lifetime clock for a cell would have been to trigger it at the time of division that gave birth to the cell. However, we must take into account the experimental observations that at high cell density, the differentiation of OPCs occurs earlier [42, 43]. This density-dependent mechanism is also compatible with the scenario where the cells limit their own growth by consuming the available mitogens such as PDGF [19, 44]. Therefore, in our model, we decided that the clock is not triggered just after a division, but starts when the cell just had interactions with other cells, and more precisely, when at least one overlap with another cell is created. On the contrary, when a cell stops having any overlap with any other cell, the lifetime clock is reset to its initial value. In this scenario, the cells differentiate earlier at high density, which is biologically realistic. When the lifetime clock of a cell reaches the lifetime threshold, the cell disappears (which experimentally could correspond to differentiation or death).

### Ethics statement

The data on human biopsies we are using have already been published elsewhere, and we show in this article a new presentation of the data.

### Clinical data and histology

The clinical data have been presented in details in [11]. Briefly, we searched the database of a previously published study performed in the Sainte-Anne Hospital in Paris (France) and focused on patients in whom a low-grade glioma was newly diagnosed by MRI-based serial stereotactic biopsies according to the Talairach stereotactic method between January 1992 and December 2001. These patients gave their informed consent for storage of the surgical samples for further analyses. We retrospectively selected nine cases of untreated adult patients. We analyzed 44 biopsy samples. After surgery, the biopsy samples were fixed in formalin-zinc and individually embedded in paraffin. Serial sections were cut at 6 *μm*. Sections used for immunohistochemistry were microwaved in citrate buffer (pH = 6) for antigen retrieval (Micro MedMicro MEDT, Hacker instruments, Winnsboro, SC) at 98deg C for 30 minutes. Ki-67 (MIB-1) immunostaining revealed MIB-1 positive cells (i.e., cycling cells).

## Results

In the initial state, one cell is placed at random in space, its lifetime clock set to zero. There is plenty of room for proliferation, so cells divide and the cell density increases. When one cell happens to have one or more overlap with other cells, its lifetime clock is triggered and increases at each following iteration. When the clock reaches the lifetime threshold, the cell disappears. With this model, the different characteristics of OPC dynamics could be recreated.

### Maintaining a constant density

**Fig 2:**
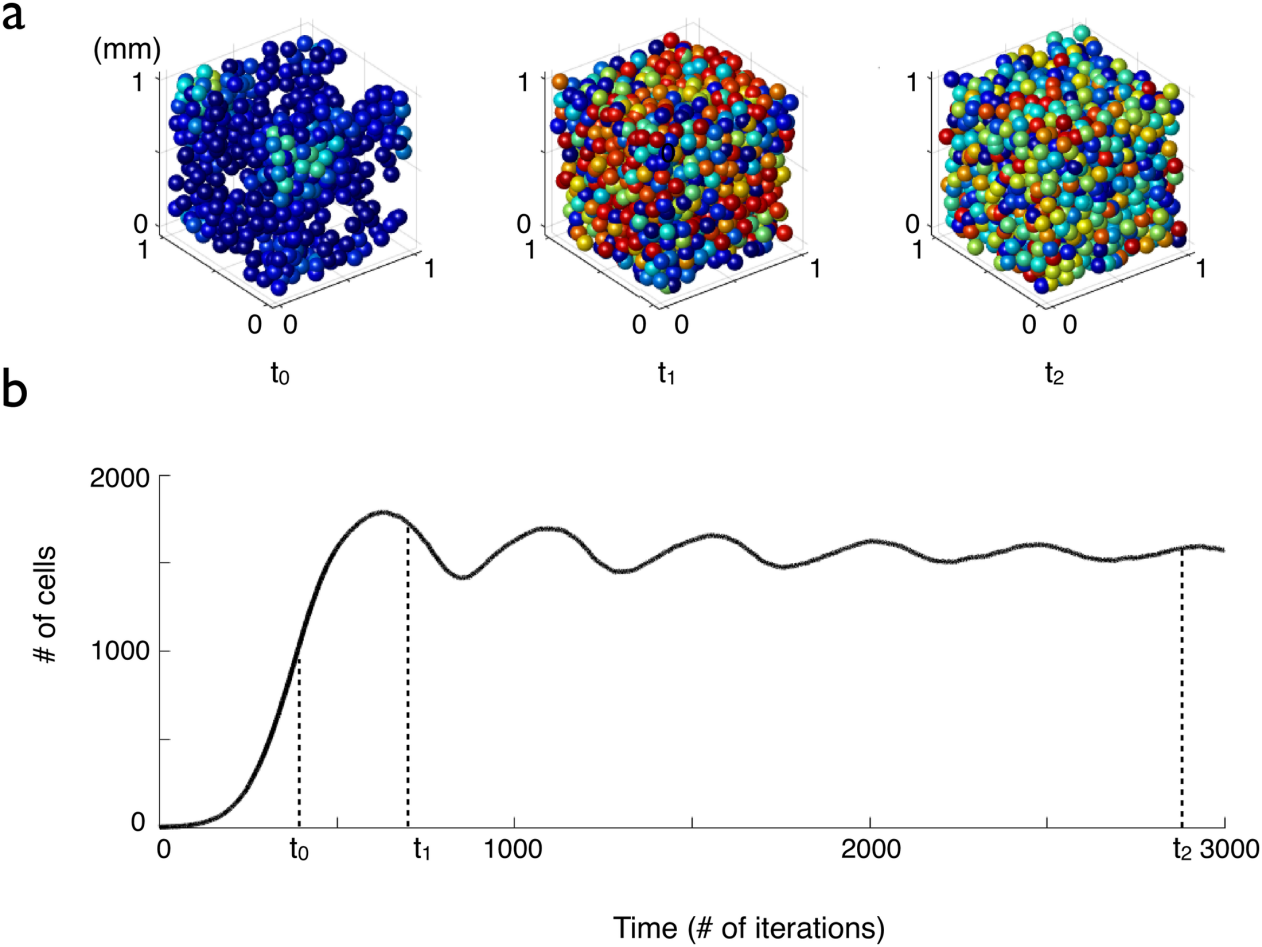
Evolution of the cell density versus time for cells with a fixed lifetime clock threshold. The proliferation parameter is λ = 0.05 per iteration and the lifetime threshold is *D* = 400 iterations. (a) The cells are represented by spheres whose color is correlated to the value of their lifetime clock: blue cells have been created recently and have a low lifetime clock, whereas red cells are close to the lifetime threshold. (b) Cell number versus time (average over 10 simulations, the error bars are smaller than the thickness of the line).

When starting with a small number of cells, after a transient state characterized by the appearance of damped oscillations, the cell number stabilises, see Fig 2(b). As in experiments, in our model the cell density reaches a constant value. The equilibrium is a dynamical one since there is still proliferation and differentiation. When a cell disappears, a neighboring cell will move and proliferate in order to fill the gap and to maintain a constant density. The cell density at equilibrium depends on the value of the lifetime threshold and on the value of the proliferation coefficient as will be explained later with a simplified model.

### Oscillations in the transient state

The characteristic time of the sharp increase in density at the beginning of each simulation (due to proliferation) is 1/λ. As the density increases, the overlapping between cells increases. This has two consequences: first, the rate of proliferation decreases and, second, many lifetime clocks are triggered. After being triggered, the clocks are increased by a unit at each iteration, and when a clock reaches the lifetime threshold, its cell disappears, see Fig 2(a). The interplay between proliferation and delayed differentiation leads to oscillations before the cell density reaches a steady state. The period of the oscillations and the coefficient of attenuation depend on the lifetime threshold and on the proliferation coefficient.

### A simplified model

In order to study these oscillations, we introduce a simplified version of the model, aiming at keeping only the characteristic features that lead to oscillations in the model, but also in order to facilitate the derivation of the mean field approximation. In this case, the three dimensional space is discretised into sites. Each cell occupies only one site (the cells are point-wise in this version). At each iteration, each cell can undergo either proliferation or differentiation/death. The proliferation rule is the same as in the full model. Since cells are point-wise, overlaps are not possible anymore. Therefore, the density-dependent differentiation rule becomes: if one or more neighboring sites of the cell are occupied, the lifetime clock is triggered.

We can calculate the mean field approximation of this simplified model. We first define *w*(*t*) the mean cell density that corresponds to the density of occupied sites in the automaton. We also define *c*(*t*) the density of sites full of cells that haven’t triggered their clock yet. There is no cell motion in this simplified model. We show that the temporal evolution of the two cell densities *c*(*t*) and *w*(*t*) satisfies respectively Eqs (1) and (2), where *D* is the lifetime threshold, λ is the proliferation coefficient, and *τ* is the time calibration parameter, see S1 Appendix:

$$\begin{array}{c} \frac{dc(t)}{dt}=\lambda w\left(t\right)[\left(1-w\left(t\right)\right]-\tau w\left(t\right)c\left(t\right) \end{array}$$ (1)

$$\begin{array}{c} \frac{dw(t)}{dt}=\lambda w\left(t\right)[\left(1-w\left(t\right)\right]-\tau w\left(t-D\right)c\left(t-D\right) \end{array}$$ (2)

The study of the steady state is detailed in S1 Appendix. To summarise, we show that in the approximation $D\gg \frac{1}{\lambda }\gg 1$ the steady state is:

$$\begin{array}{c} w_{\infty }\approx 1-\frac{1}{\lambda D}-\frac{\tau }{\lambda D^{2}} \end{array}$$ (3)

and

$$\begin{array}{c} c_{\infty }\approx \frac{\tau }{D}-\frac{\tau }{\lambda D^{2}} \end{array}$$ (4)

The study of stability is also developed in S1 Appendix. We show that the period *T* of the damped oscillations is given by the following expression:

$$\begin{array}{c} T=\frac{D}{1-\frac{1}{\lambda D}} \end{array}$$ (5)

When λ*D* ≫ 1, we can further reduce the expression of the period to: *T* ≈ *D*. In S1 Fig, the period *T* either calculated with the analytical expression (plain line), measured in simulations with the simplified cellular automaton with point-wise cells or measured in simulations with the cellular automaton with spheres, is plotted as a function of lifetime threshold *D* and as a function of the proliferation coefficient λ.

### Increasing the damping coefficient

In [45, 46], the authors report that after a lesion, the cell density in the lesion increases, displays an overshoot and then reaches its equilibrium value, without any sustained oscillations. In our model, as we showed before, if the cells have a fixed value of the lifetime duration, the system that has been brought far form equilibrium by a lesion for exemple, comes back to equilibrium, with several oscillations. One possible simple way to increase the damping coefficient of the oscillations is to broaden the distribution of lifetime thresholds. We thus choose the lifetime threshold of each cell *D* in a shifted exponential distribution of lifetimes:

$$\begin{array}{c} D=D_{0}-D_{1}\ln\left(1-r\right) \end{array}$$

where *r* is a random number uniformly between 0 and 1 and *D*_0_ and *D*_1_ are two parameters that characterize respectively the minimum lifetime threshold and the width of the distribution.

### Comparison with experimental data: Choice of parameters, time and space scale calibration

Since there is no underlying spatial network in the sphere model, the spatial length that will serve as a reference is the radius of a cell, which, experimentally [5], has been found to be around 50 *μ*m. The total volume we consider in the simulations is the volume of a cube of 20-30 cell radii.

In the automaton, time is measured as a number of iterations. In order to compare the simulation results with the experimental ones, a calibration of the time scale is necessary by defining the duration of one iteration in a physical time unit. We have chosen one iteration of the automaton to represent 1 h.

Two parameters have to be fixed in the simulations: the proliferation coefficient and the lifetime threshold parameter. For the choice of these parameters, we have a first constraint on the lifetime threshold. The latter must be larger than the duration of the cell cycle so that the cells have time to proliferate several times before dying (λ*D* ≫ 1). This condition allows to reach the cell density observed in experiments.

In order to fine-tune the values of the time-related parameters we review what is experimentally known, in rodents. Both [45] and [46] present results of the order of magnitude of the time necessary to close a lesion. In these articles, toxin-induced focal lesions are caused in rat brain with ethidium bromide. This drug is cytotoxic and kills all nucleated cell types, creating a lesion of around 500 *μ*m diameter. Right after the lesion, the cells at the border proliferate more and migrate. The cell density inside the lesion increases sharply, overshoots the normal density, reaches a maximum after ten days and finally goes back to normality after 15-30 days. As the time to return to equilibrium is close to the value of the mean lifetime threshold (in the case λ*D* ≫ 1), it means that the value of *D*_0_ should be close to 20 days. The second temporal order of magnitude can be found in [5], where the cycle duration of the OPCs was measured in vivo in mice at around 20 days.

We found that the parameters λ = 0.05 h^−1^, *D*_0_ = 25 days and *D*_1_ = *D*_0_/2 are a very good compromise between the different constraints. We will now present the results of the simulations that can be compared with the experimental data. The simulation presented at first is the no-lesion case.

**Fig 3:**
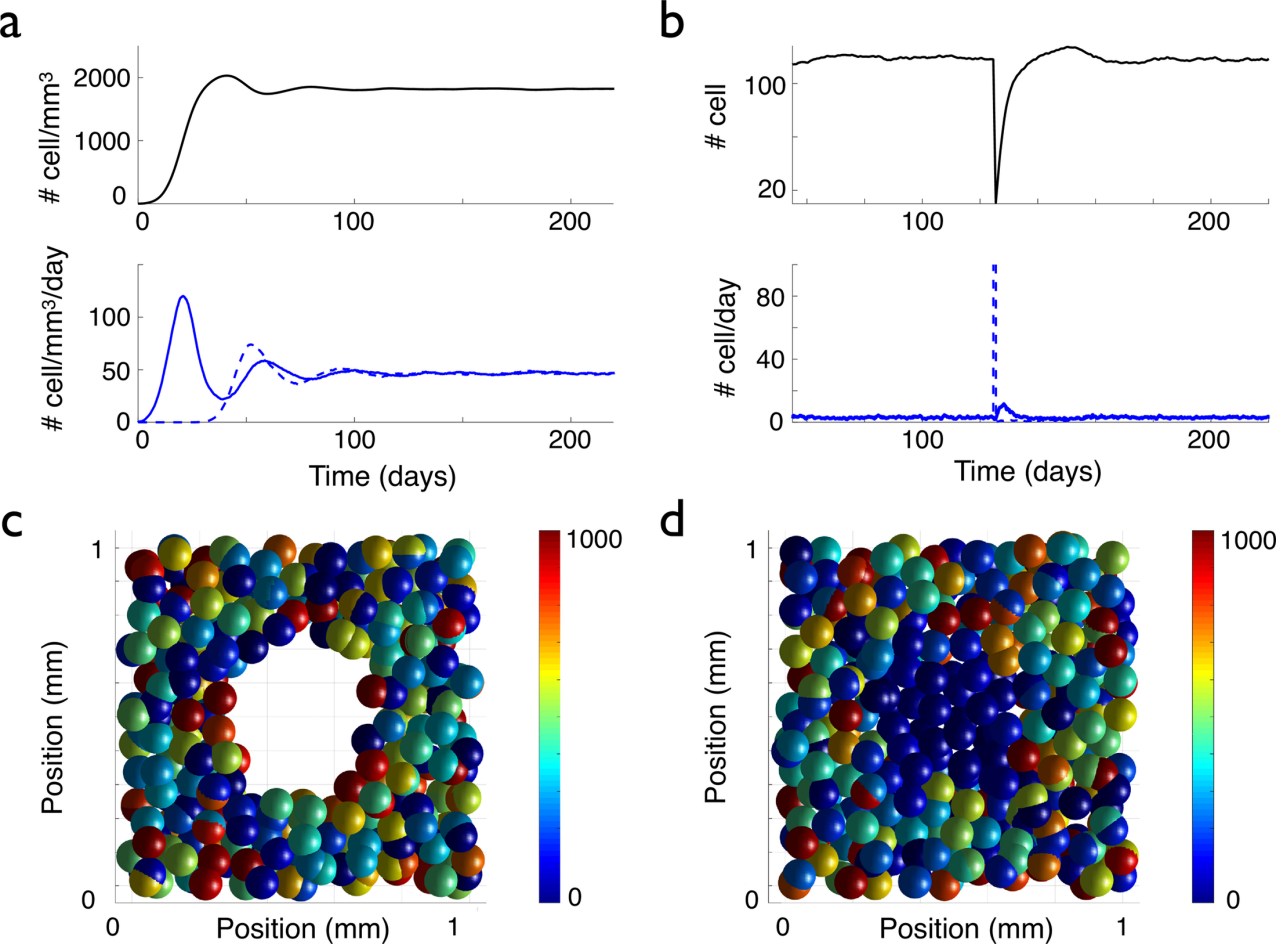
Evolution of the cell density, with and without a lesion. The graphs in (a) display the temporal evolution of the cell density (up) and the density per day of proliferating (bottom, blue curve) and differentiating (bottom, red curve) cells, when starting the simulation with one cell. In (b), a lesion is made at time *t* = 125 days: all the cells inside a sphere of 250 *μ*m radius centered at the center of space, are killed (see (c)). The graphs in (b) display the temporal evolution of the cell number (up) and the numbers of proliferating (bottom, blue plain curve) and disappearing (bottom, blue dashed curve) cells per day inside the sphere corresponding to the initial lesion, see (c) (average over 20 simulations, the error bars are not represented to avoid overloading the figure but they can be estimated from the amplitude of the fluctuations). In (c) and (d), the color of the cells is correlated with the value of their lifetime clock (with a maximum lifetime threshold of 1000 h). In (c) and (d), in order to be able to see the lesion, only a 200 *μ*m thick slice centered at the origin is represented. In (c), the system is represented just after the 250 *μ*m radius lesion. (d) The lesion is filled up after 33 days of evolution by the migration and the proliferation of the cells at the border of the lesion. The newly formed cells or migrating cells that have reset their clock by loosing their contact with the neighboring cells inside the perimeter of the lesion appear in blue.

In the simulations, we start with one cell so we first have to wait for the system to reach its equilibrium point. In Fig 3(a), the total cell density in space is represented. The first remark is that the oscillations are highly damped (as expected) when the total cell density converges towards its equilibrium value at the beginning of the simulation for times *t* < 80 days. At equilibrium, in the total volume of simulation (a cube of 1 mm^3^) there are around 2000 cells, meaning that the cell density is equal to 2000 cells per mm^3^, see Fig 3(a). In experiments, 160 cells were counted in a volume of 0.06 mm^3^, so the experimental density is approximately equal to 2700 cell/mm^3^ [5]. The two values agree well.

Also, in the equilibrium state, other variables have the same order of magnitude as in experiments:

- The mean cell velocity: the mean velocity of cells measured over 40 days in simulations is equal to 0.13 (±0.03) *μ*m/iteration, or 3.16 (±0.07) *μ*m/day, which is close to the experimental mean velocity of 2.3 *μ*m/day.
- The fraction of proliferating cells: the number of proliferating cells is also very close to the experimental result. We find that in the simulations, 1.6% of the cells proliferate each day, (see Fig 3(a) where the dashed blue curve represents the proliferating cells), which is similar to the 1.5% per day measured experimentally. Actually, most of the cells differentiate or die before dividing even once.
- The mean time between two divisions, i.e. the mean duration of cell cycle, measured during 40 days at equilibrium, is equal to 12 days ±0.5 in the simulations, also close to the experimental result of 20 days.

We also simulated the repopulation after a lesion killed all the cells in an area. At time *t* = 125 days after the beginning of the simulation, a lesion of 500 *μ*m diameter is induced see Fig 3(c). In the total cell density (see Fig 3(b)), the lesion is materialized by a peak in the dying/differentiating cells (dashed blue curve), followed by another peak in the proliferating curve (plain blue curve) when the cells at the border of the lesion begin to replenish the empty space (see also S1 Video). The cell density inside the area of the initial lesion overshoots and goes back to its equilibrium value with further oscillations 40 days after the induction of the lesion, see Fig 3(b). This duration is compatible with the experimental estimates. Fig 3 shows the system of cells at the time of the lesion (c) and after 6 days of evolution (d), when the empty space of the lesion has been filled by new cells (appearing in blue on the figure, meaning that their lifetime clock is low), created by the proliferation and the migration of the cells at the border of the lesion. This behaviour mimics very well what happens in the experiments, where the induction of proliferation and migration of cells around a lesion has been also observed [5].

### Going to glioma

**Fig 4:**
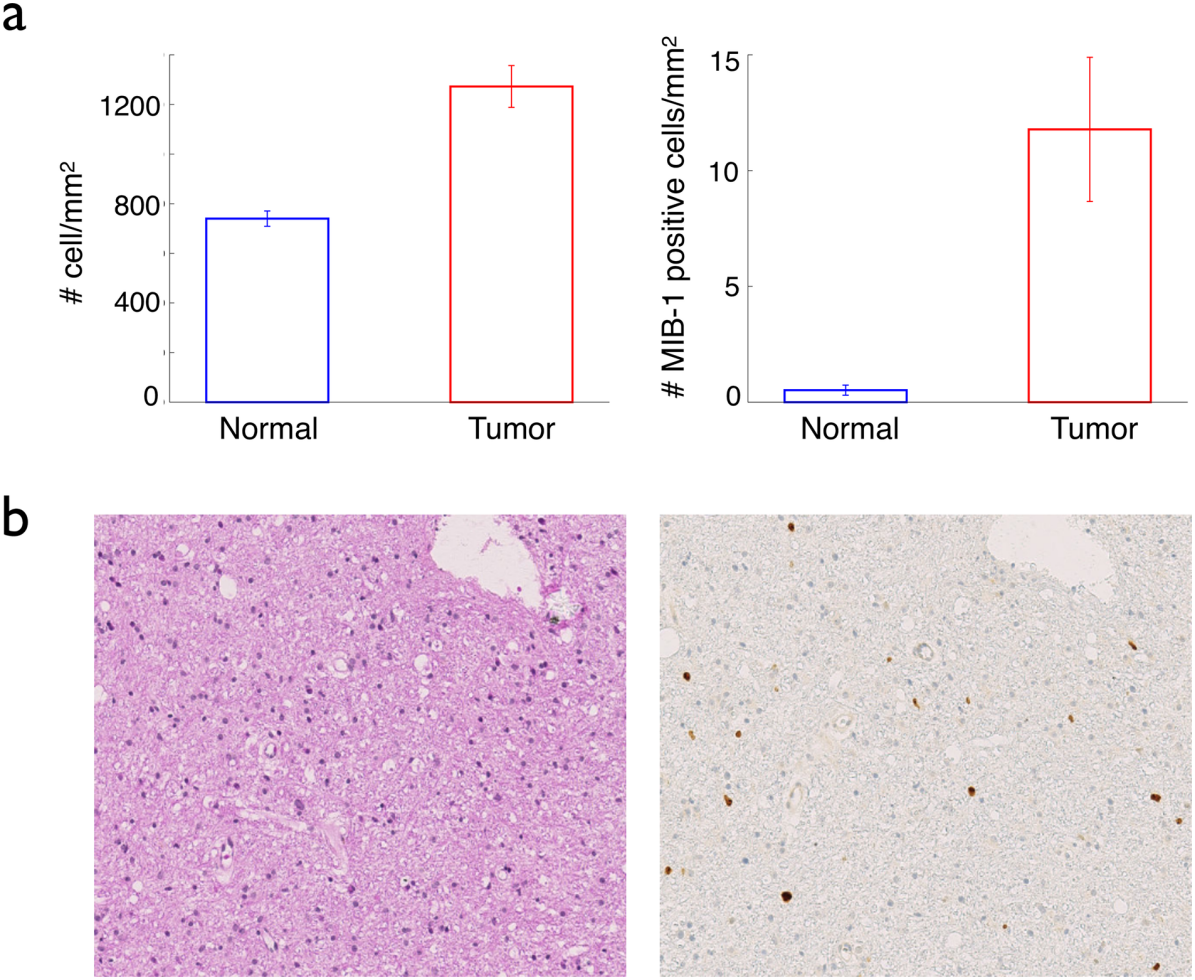
Total and proliferating cell densities in real gliomas. The graphs in (a) compare the mean total cell density (left) and the mean MIB-1 positive cell density (the MIB-1 positive cells are the cells that have entered the cell cycle, i.e. the proliferating cells) (right), inside (red bars) and outside (blue) real gliomas. The data come from 9 different patients, 22 samples inside the tumor (i.e. inside the signal abnormality on T2 MRI scans) and 16 samples ouside. In (b) left, a histological sample of a low-grade glioma, with a hematoxylin-eosin staining, displays a quasi-normal cell density. In (b), right, the same sample stained with the proliferation staining MIB-1, reveals a limited increase in the proliferating cell density compared to normal tissue. The detailed data have been published in [11].

In this section, we explored the possibility that in our simulations, the appearance of a cell with only one different property than the existing cells could lead to the formation of a glioma. We compared the results of the simulations in each cases with the properties of real low-grade gliomas: a) there is a limited increase in the total and in the proliferating cell density in the tumor; b) the radius of the tumor increases linearly, with a low velocity and c) the tumor is invasive with diffuse boundaries. Concerning the first point, we had the opportunity to have access to stereotactic biopsies from different spots of the same human tumor and we showed that in the center of the tumor, the cell density was higher than that of the surrounding normal tissue, but only by a factor close to 2 [11] (up to a factor of three larger in some cases, but on average 34 samples, a factor of 1.7), see Fig 4(a).

**Fig 5:**
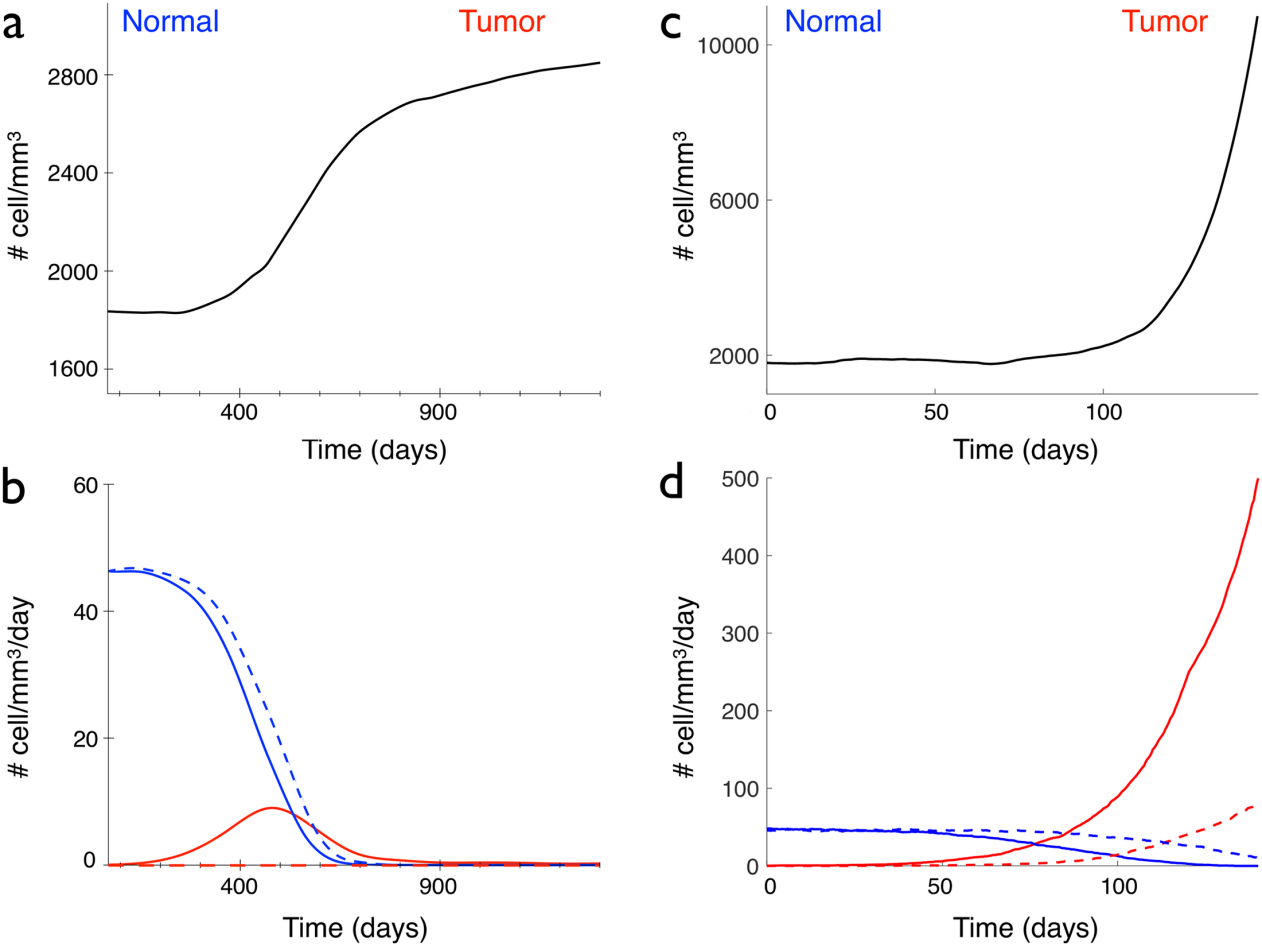
Comparison of the different scenarios of glioma appearance. (a) and (b), an immortal cell appears at time *t* = 0, (c) and (d), a cell without contact inhibition appears at time *t* = 0. (a) and (c) Temporal evolution of the total cell density. (b) and (d) Normal (blue lines) and tumoral (red lines) proliferating (plain curve) and disappearing (dashed curve) cell densities, in a 1 mm^3^ cube.

We studied three possible scenarios for the genesis of a glioma: the appearance of either an immortal cell, or a contact-inhibition free or an over proliferating cell, that transmits its abnormal property to its daughter cells. In the simulations, this abnormal original cell corresponds to a given number fixed at the beginning of the simulation (for exemple the 2000th cell). We vary only one parameter at a time. The first scenario that we studied is the acquisition of a lifetime advantage by the abnormal original cell: its lifetime clock threshold is longer than the duration of simulations, so that the cell cannot die during the simulation. All its other properties are maintained the same (proliferation, migration, contact inhibition). All its daughter cells keep the same property. Therefore, normal dying cells are progressively replaced by these immortal cells. A tumor forms and the cell density inside the tumor reaches a new equilibrium at a larger value than in the normal case, see Fig 5(a). This is in good agreement with Eq (3) that tells us that the cell density at equilibrium increases with the lifetime. However, since the new cells do not die, the density of proliferating cells decreases down to zero, see Fig 5(b). This feature is not consistent with the histological samples and the experimental observations on low-grade gliomas (see Fig 4(a) and 4(b), and in [11]) where the proliferation inside the tumor is low but higher than in the normal tissue.

The second scenario that we studied is the bypass of the saturation of the proliferation rule. This rule stipulates that a cell 1 can proliferate only if the distances between the newly created cell and all the cells that overlap cell 1 are larger than the cell radius *R*. The abnormal original cell and its daughters are thus not limited in their proliferation, so the cell density increases very fast, see Fig 5(c) and 5(d). A tumor forms again but in contrast with the previous scenario, no new equilibrium of the cell density inside the tumor is reached. The cell density explodes and the situation is be closer to what happens in high-grade gliomas (where cells at the center of the tumor are closely packed), than to low-grade gliomas. In order to keep a cell density reasonable, as in low-grade gliomas, the saturation-of-proliferation rule seems to be necessary.

**Fig 6:**
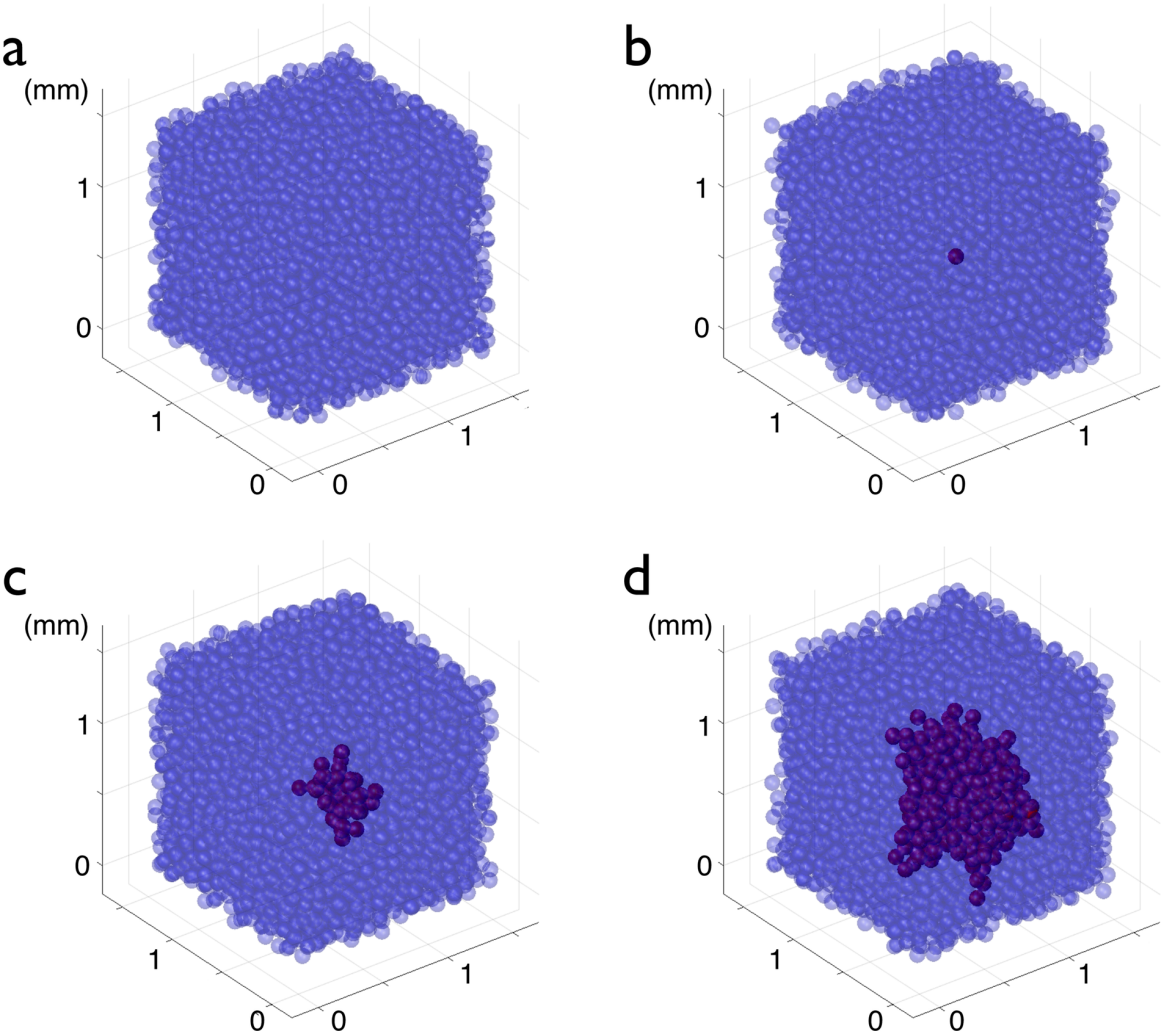
Formation of a glioma by the appearance of an over proliferating cell. (a) Normal OPCs (blue) at equilibrium proliferate (*ρ* = 0.05/h) and differentiate, as described in the text. In (b), a newly created cell is characterized by an over-proliferating (*ρ* = 0.25/h) phenotype, in red (*t* = 0). The daughters of this abnormal cell keep the over-proliferating character. In (c) the system is represented at *t* = 1500 h = 62.5 days, the developing glioma appears in dark red; in (d) the system is represented at *t* = 3000 h = 125 days.

The last scenario we studied was the appearance of an abnormal cell with a higher proliferation coefficient. This abnormal original cell is associated with a proliferation coefficient five times higher (0.25 h^−1^) than the proliferation coefficient of all the other cells around it (0.05 h^−1^). All the progeny of this mutated cell has the same high proliferation coefficient. The holes left by normal cells that die are quickly filled by highly-proliferating ones. As the small highly-proliferating core progressively grows, glioma and normal cells compete for space. Fig 6 depicts the temporal evolution of such a glioma (see also S2 Video).

**Fig 7:**
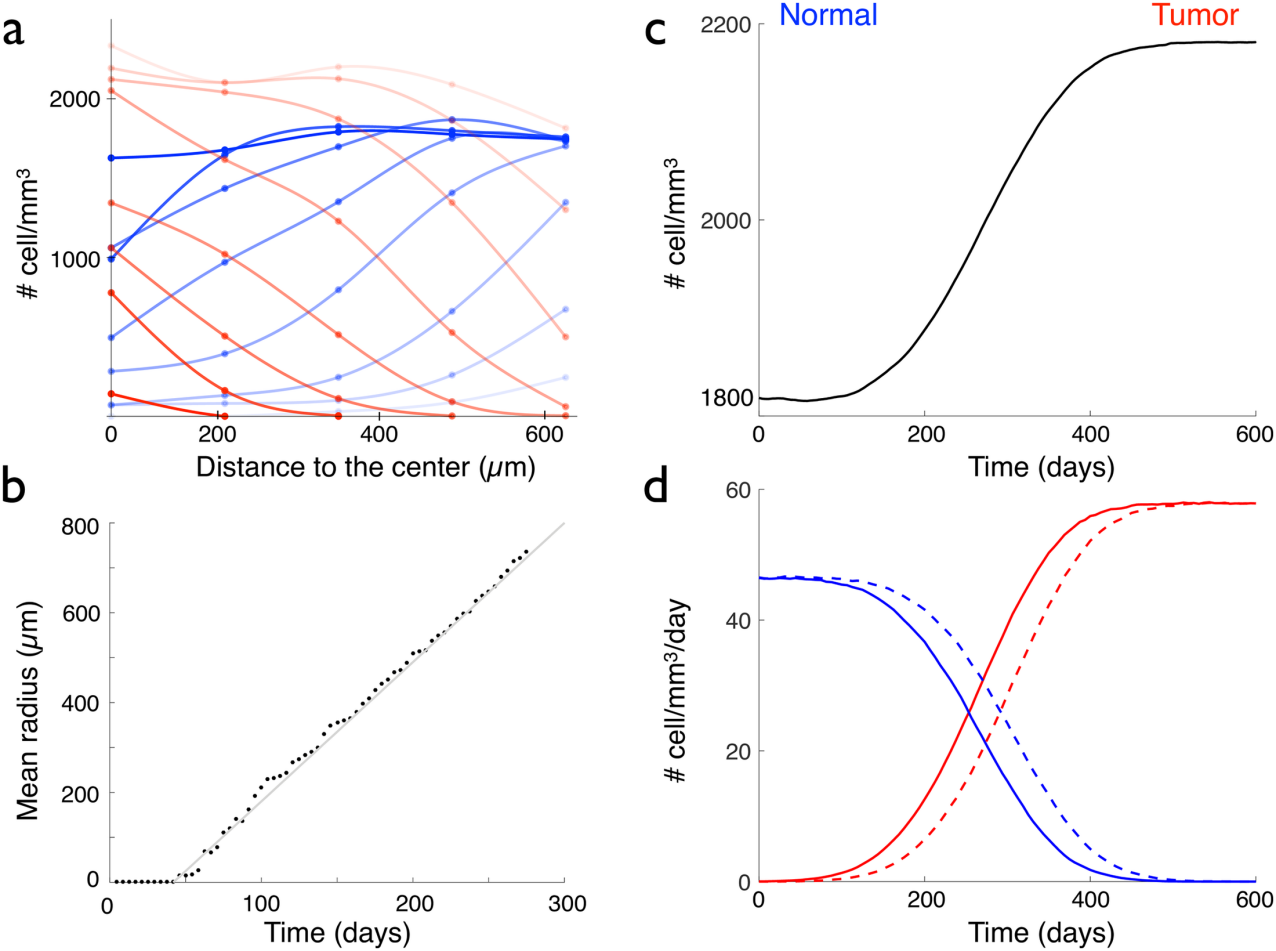
Properties of a glioma formed by the appearance of an over proliferating cell. (a) Normal (blue circles) and glioma (red circles) cell densities versus the distance to the center of space, for the glioma of Fig 6. Eight graphs corresponding to eight time points are represented from *t* = 0 (appearance of the first glioma cell, dark red and blue graphs), to *t* = 336 days (very light red and blue graphs), with a time interval of 42 days. (b) Temporal evolution of the mean radius of the glioma of Fig 6. (c) Temporal evolution of the total cell density, when an over proliferating cell appears at time *t* = 0. (d) Normal (blue lines) and tumoral (red lines) proliferating (plain curve) and disappearing (dashed curve) cell densities, in a 1 mm^3^ cube, where an over proliferating cell appears at time *t* = 0.

Where both cells types cohabit, Fig 7(a) reveals that normal cell density decreases with time (blue circles and lines), whereas the glioma cell density increases (red circles and lines). After a long time, the normal cells disappear and all the cells bear the mutation. In the tumor, the over proliferating cells reach a new equilibrium: the cell density is constant and higher than before, as predicted by Eq (3) and as it can be observed in Fig 7(c).

Since the third scenario (an over-proliferating cell) corresponds to the better agreement with the cell density and proliferating cell density observed in real low-grade gliomas, we kept only this scenario for further comparisons with clinical properties of gliomas.

As real low-grade gliomas, the simulated tumour is invasive: the border of the tumor are not regular and the tumor cell density decreases progressively from the center to the normal tissue, see Figs 6 and 7(a). Since the normal cells already have migration properties, there is no need in our model to introduce specific migration properties for tumor cells in order to obtain an invasive tumor. Moreover, the evolution of the mean radius of the core in the three cartesian directions is plotted and fitted, see Fig 7(b). We find that the growth of the core is linear, and the velocity of growth of this core in the simulations is around 1.1 mm/yr.

## Discussion

In this article, we present a model of oligodendrocyte precursor cells dynamics. The model is based on a cellular automaton and the rules that we define are close to the biological reality: the virtual cells can divide, disappear as OPCs (either differentiate or die) after a given lifetime that depends on the local cell density, and move in the direction that will minimise their overlaps with other cells. Our model reproduces the main properties of the OPCs that have been described experimentally in a normal tissue: it allows to achieve the same homeostasis with a constant density and the same behavior after a lesion when the surrounding cells proliferate and migrate in order to fill the injured area. Our model also achieves a very good quantitative agreement with experiments in rodents, since the theoretical and experimental cell density, mean velocity, cycle duration, fraction of proliferating cells are very similar.

There is a strong suspicion that OPCs and more generally glial progenitor cells are at the origin of gliomas [19, 20, 22]. As OPCs retain some of their characteristics from development to adulthood, including the ability to proliferate and migrate, they could easily transform to an uncontrolled growth state. We proposed three different scenarios for the genesis of gliomas: the appearance of a deadless cell, a cell that has lost its contact inhibition for proliferation and an over proliferating cell. The deadless cells could also correspond to non-differentiating cells, since in our simulations, we do not separate death from differentiation. Those three scenarios are not the only ones possible for gliomatogenesis, but are among the most probable [47]. we found that these three scenarios lead to the formation of gliomas, but with very different characteristics:

- the scenario of the deadless/non-differentiating cell: this scenario can occur at an early stage of the glioma development but is not sufficient to form a tumor that has the same properties as the real gliomas. This result is surprising and goes against the conclusion of other models where escape from differentiation is the way to form tumors [48]. Such a scenario may have been observed in experiments. In [22], mutations (p53/NF1) are triggered in a population of OPCs in mouse adult brain by the injection of a specific drug. A few days after the injection, an transient increase of proliferation of mutated OPCs is detected, corresponding to an impairment of the differentiation of the mutated cells, but soon their proliferation returns to the basal level. After a phase of quiescence, these cells suddenly begin to over-proliferate and form eventually a malignant glioma.
- the scenario of the loss of contact inhibition: this scenario leads to the very fast formation of a glioma characterized by a very high total and proliferating cell densities inside the tumor, closer to a high-grade glioma. This maybe the second step of transformation of OPCs in [22], after the phase of quiescence, leading to the formation of a malignant glioma.
- the scenario of the appearance of an over proliferating cell: as in the scenario of the deadless cell, the glioma cell density inside the tumor reaches an equilibrium when highly-proliferating cells have replaced normal ones. This equilibrium is characterized by a limited increase in the total and proliferating cell densities, compared to normal tissue. From comparison with histological samples of real gliomas, we can conclude that the scenario of the over proliferating cell as cell of origin is the one that reproduces the best the characteristics of low-grade gliomas. This new equilibrium could explain why low-grade gliomas may remain indolent during one or more decades [13]. The anaplastic transformation of low towards higher grade glioma could thus correspond to the departure from this equilibrium, by the appearance of a new mutation or by the pressure of the microenvironment [49]. Since our model focuses on the origin of gliomas, it does not account for the progression of low-grade into secondary high-grade gliomas.

Another feature of real gliomas that is very well reproduced by our *in silico* glioma is the linear growth of the tumor radius. Actually, this property of the tumor could have been predicted: as long as the rules of the automaton include linear proliferation and migration, the tumor is invasive and the model naturally reproduces the linear increase of the radius of the tumor at large time, observed clinically. This linear behaviour of the tumor radius is the same as the constant velocity observed by the propagating front obtained with the reaction-diffusion model used for gliomas [50]. Here, in the scenario of the appearance of an over-proliferating cell, we measured that the velocity of increase of the tumor radius is around 1.1 mm/yr., which is close to the mean velocity measured for human low-grade gliomas of 2 mm/yr [14]. However, this quantitative agreement has to be taken with a grain of salt since the model has been calibrated with mouse data. The proliferation coefficient and lifetime clock may not be the same in humans.

Our model shows that the dynamics of OPCs is compatible with the fact that they can be at the origin of gliomas. We provide different scenarios that could lead to the formation of low-grade and high-grade gliomas. Challenging this result with clinical data from histology will constitue the objective of our future work.

## Supporting information

S1 Appendix Analysis of the oscillations. (PDF)

S1 Fig Period of oscillations versus lifetime clock threshold and the proliferation coefficient. Period of oscillations versus the lifetime clock threshold (black crosses, line and full circles) and the proliferation coefficient (gray crosses, line and open circles), calculated from the analytical formula (5) (lines), from the simplified cellular automaton with point-wise cells (crosses) and from the cellular automaton with spheres (circles).
(PDF)

S1 Video Closure of a lesion. The color of the cell is related to the value of its lifetime clock. A cell with a low lifetime clock appears in blue, whereas a cell with a lifetime clock close to the threshold is red.
(AVI)

S2 Video Formation of a glioma by the appearance of an over proliferating cell. The normal cells are blue and the over-proliferating cells are red.
(AVI)

## References

[1] Geha S, Pallud J, Junier MP, Devaux B, Leonard N, Chassoux F, et al NG2+/Olig2+ cells are the major cycle-related cell population of the adult human normal brain. Brain Pathol. 2010;20:399–411. doi: 10.1111/j.1750-3639.2009.00295.x 19486010 10.1111/j.1750-3639.2009.00295.x PMC8094800

[2] Dawson MRL, Polito A, Levine JM, Reynolds R. NG2-expressing glial progenitor cells: an abundant and widespread population of cycling cells in the adult rat CNS. Mol Cell Neurosci. 2003;24:476–488. doi: 10.1016/S1044-7431(03)00210-0 14572468 10.1016/s1044-7431(03)00210-0

[3] Nishiyama A, Suzuki R, Zhu X. NG2 cells (polydendrocytes) in brain physiology and repair. Front Neurosci. 2014;8:133 doi: 10.3389/fnins.2014.00133 25018689 10.3389/fnins.2014.00133 PMC4072963

[4] Sanai N, Tramontin AD, Quiñones Hinojosa A, Barbaro NM, Gupta N, Kunwar S, et al Unique astrocyte ribbon in adult human brain contains neural stem cells but lacks chain migration. Nature. 2004;427:740–744. doi: 10.1038/nature02301 14973487 10.1038/nature02301

[5] Hughes EG, Kang SH, Fukaya M, Bergles DE. Oligodendrocyte progenitors balance growth with self-repulsion to achieve homeostasis in the adult brain. Nat Neurosci. 2013;16:668–676. doi: 10.1038/nn.3390 23624515 10.1038/nn.3390 PMC3807738

[6] Clemente D, Ortega MC, Melero-Jerez C, de Castro F. The effect of glia-glia interactions on oligodendrocyte precursor cell biology during development and in demyelinating diseases. Front Cell Neurosci. 2013;7:268 doi: 10.3389/fncel.2013.00268 24391545 10.3389/fncel.2013.00268 PMC3868919

[7] Schwartzbaum JA, Fisher JL, Aldape KD, Wrensch M. Epidemiology and molecular pathology of glioma. Nat Clin Pract Neuroll. 2006;2:494–503. doi: 10.1038/ncpneuro0289 10.1038/ncpneuro0289 16932614

[8] Stupp R, Mason WP, van den Bent MJ, Weller M, Fisher B, Taphoorn MJ, et al Radiotherapy plus concomitant and adjuvant temozolomide for glioblastoma. N Engl J Med. 2005;352(10):987–996. doi: 10.1056/NEJMoa043330 15758009 10.1056/NEJMoa043330

[9] Louis DL, Ohgaki H, Wiestler OD, Cavenee WK, Burger PC, Jouvet A, et al The 2007 WHO Classification of Tumours of the Central Nervous System. Acta Neuropathologica. 2007;114(2):97–109. doi: 10.1007/s00401-007-0243-4 17618441 10.1007/s00401-007-0243-4 PMC1929165

[10] Pallud J, Varlet P, Devaux B, Geha S, Badoual M, Deroulers C, et al Diffuse low-grade oligodendrogliomas extend beyond MRI-defined abnormalities. Neurology. 2010;74:1724–1731. doi: 10.1212/WNL.0b013e3181e04264 20498440 10.1212/WNL.0b013e3181e04264

[11] Gerin C, Pallud J, Deroulers C, Varlet P, Oppenheim C, Roux FX, et al Quantitative characterization of the imaging limits of diffuse low-grade oligodendrogliomas. Neuro-Oncology. 2013;15:1379–1388. arXiv:1803.09005 [q-bio.TO] doi: 10.1093/neuonc/not072 23771168 10.1093/neuonc/not072 PMC3779035

[12] Pallud J, Fontaine D, Duffau H, Mandonnet E, Sanai N, Taillandier L, et al Natural history of incidental WHO grade II gliomas. Annals of Neurology. 2010;68:727–33. doi: 10.1002/ana.22106 21031584 10.1002/ana.22106

[13] Gerin C, Pallud J, Grammaticos B, Mandonnet E, Deroulers C, Varlet P, et al Improving the time-machine: estimating date of birth of grade II gliomas. Cell Prolif. 2012;45:76–90. doi: 10.1111/j.1365-2184.2011.00790.x 22168136 10.1111/j.1365-2184.2011.00790.x PMC6496223

[14] Mandonnet E, Delattre JY, Tanguy ML, Swanson KR, Carpentier AF, Duffau H, et al Continuous growth of mean tumor diameter in a subset of grade II gliomas. Annals of Neurology. 2003;53(4):524–528. doi: 10.1002/ana.10528 12666121 10.1002/ana.10528

[15] Pallud J, Mandonnet E, Duffau H, Kujas M, Guillevin R, Galanaud D, et al Prognostic value of initial magnetic resonance imaging growth rates for World Health Organization grade II gliomas. Ann Neurol. 2006;60:380–383. doi: 10.1002/ana.20946 16983683 10.1002/ana.20946

[16] Prabhu VC, Khaldi A, Barton KP, Melian E, Schneck MJ, Primeau MJ, et al Management of diffuse low-grade cerebral gliomas. Neurologic Clinics. 2010;28(4):1037–1059. doi: 10.1016/j.ncl.2010.03.022 20816276 10.1016/j.ncl.2010.03.022

[17] Stallcup WB, Huang FJ. A role for the NG2 proteoglycan in glioma progression. Cell Adh Migr. 2008;2:192–201. doi: 10.4161/cam.2.3.6279 19262111 10.4161/cam.2.3.6279 PMC2634088

[18] Persson AI, Petritsch C, Swartling FJ, Itsara M, Sim FJ, Auvergne R, et al Non-stem cell origin for oligodendroglioma. Cancer Cell. 2010;18:669–682. doi: 10.1016/j.ccr.2010.10.033 21156288 10.1016/j.ccr.2010.10.033 PMC3031116

[19] Bergles DE, Richardson WD. Oligodendrocyte Development and Plasticity. Cold Spring Harb Perspect Biol. 2015;8:a020453 doi: 10.1101/cshperspect.a020453 26492571 10.1101/cshperspect.a020453 PMC4743079

[20] Canoll P, Goldman JE. The interface between glial progenitors and gliomas. Acta Neuropathol. 2008;116:465 doi: 10.1007/s00401-008-0432-9 18784926 10.1007/s00401-008-0432-9 PMC2759726

[21] Liu C, Sage JC, Miller MR, Verhaak RGW, Hippenmeyer S, Vogel H, et al Mosaic Analysis with Double Markers (MADM) Reveals Tumor Cell-of-Origin in Glioma. Cell. 2011;146:209–221. doi: 10.1016/j.cell.2011.06.014 21737130 10.1016/j.cell.2011.06.014 PMC3143261

[22] Galvao R, Kasina A, McNeill R, Harbin J, Foreman O, Verhaak R, et al Transformation of quiescent adult oligodendrocyte precursor cells into malignant glioma through a multistep reactivation process. Proc Nat Acad Sci USA. 2014;111(40):E4214–E4223. doi: 10.1073/pnas.1414389111 25246577 10.1073/pnas.1414389111 PMC4210043

[23] Altrock PM, Liu LL, Michor F. The mathematics of cancer: integrating quantitative models. Nat Rev Cancer. 2011;15:730–745. doi: 10.1038/nrc4029 10.1038/nrc4029 26597528

[24] Spencer SL, Berryman MJ, Garcia JA, Abbott D. An ordinary differential equation model for the multistep transformation to cancer. J Theor Biol. 2004;231:515–524. doi: 10.1016/j.jtbi.2004.07.006 15488528 10.1016/j.jtbi.2004.07.006

[25] Beerenwinkel N, Antal T, Dingli D, Traulsen A, Kinzler KW, Velculescu VE, et al Genetic progression and the waiting time to cancer. PLoS Comput. 2007;3:e225 doi: 10.1371/journal.pcbi.0030225 10.1371/journal.pcbi.0030225 PMC2065895 17997597

[26] Tomlinson IP, Bodmer WF. Failure of programmed cell death and differentiation as causes of tumors: some simple mathematical models. Proc Natl Acad Sci U S A. 1995;92:11130–11134. 7479951 10.1073/pnas.92.24.11130 PMC40585

[27] Rodriguez-Brenes IA, Komarova NL, Wodarz D. Evolutionary dynamics of feedback escape and the development of stem-cell-driven cancers. Proc Natl Acad Sci U S A. 2011;108:18983–18988. doi: 10.1073/pnas.1107621108 22084071 10.1073/pnas.1107621108 PMC3223454

[28] H Y, Li X, Lander AD, Lowengrub JS. Multispecies model of cell lineages and feedback control in solid tumors. J Theor Biol. 2012;304:39–59. doi: 10.1016/j.jtbi.2012.02.030 22554945 10.1016/j.jtbi.2012.02.030 PMC3436435

[29] Enderling H, Hlatky L, Hahnfeldt P. Cancer Stem Cells: A Minor Cancer Subpopulation that Redefines Global Cancer Features. Front Oncol. 2013;3:76 doi: 10.3389/fonc.2013.00076 23596563 10.3389/fonc.2013.00076 PMC3625721

[30] Aubert M, Badoual M, Féreol S, Christov C, Grammaticos B. A cellular automaton model for the migration of glioma cells. Phys. Biol. 2006;3:93–100. doi: 10.1088/1478-3975/3/2/001 16829695 10.1088/1478-3975/3/2/001

[31] Kavousanakis ME, Liu P, Boudouvis AG, Lowengrub J, Kevrekidis IG. Efficient coarse simulation of a growing avascular tumor. Phys Rev E Stat Nonlin Soft Matter Phys. 2012;85:031912 doi: 10.1103/PhysRevE.85.031912 22587128 10.1103/PhysRevE.85.031912 PMC3833450

[32] Swanson KR, Bridge C, Murray JD, A EC Jr. Virtual and real brain tumors: using mathematical modeling to quantify glioma growth and invasion. Journal of the Neurological Sciences. 2003;216:289–296. doi: 10.1016/j.jns.2003.06.001 10.1016/j.jns.2003.06.001 14607296

[33] Pérez-García VM, Pérez-Romasanta LA. Extreme protraction for low-grade gliomas: theoretical proof of concept of a novel therapeutical strategy. Math Med Biol. 2017;33:253–271. 10.1093/imammb/dqv017 25969501

[34] Jackson PR, Juliano J, Hawkins-Daarud A, Rockne RC, Swanson KR. Patient-specific mathematical neuro-oncology: using a simple proliferation and invasion tumor model to inform clinical practice. Bull Math Biol. 2015;77:846–856. doi: 10.1007/s11538-015-0067-7 25795318 10.1007/s11538-015-0067-7 PMC4445762

[35] Zhang L, Wang Z, Sagotsky AJ, Deisboeck TS. Multiscale agent-based cancer modeling. J Math Biol. 2009;58:545–559. doi: 10.1007/s00285-008-0211-1 18787828 10.1007/s00285-008-0211-1

[36] Caiazzo A, Ramis-Conde I. Multiscale modeling of palisade formation in Glioblastoma Multiforme. J Theor Biol. 2005;383:145–156. doi: 10.1016/j.jtbi.2015.07.021 10.1016/j.jtbi.2015.07.021 26235287

[37] Wang Z, Butner JD, Kerketta R, Cristini V, Deisboeck TS. Simulating cancer growth with multiscale agent-based modeling. Semin Cancer Biol. 2015;30:70–78. doi: 10.1016/j.semcancer.2014.04.001 24793698 10.1016/j.semcancer.2014.04.001 PMC4216775

[38] Lander AD, Lowengrub JS. Cell lineages and the logic of proliferative control. PLoS Biol. 2009;7:e15 doi: 10.1371/journal.pbio.1000015 19166268 10.1371/journal.pbio.1000015 PMC2628408

[39] Zhang H, Miller RH. Density-Dependent Feedback Inhibition of Oligodendrocyte Precursor Expansion. J Neurosci. 1996;16:6886–6895. 8824327 10.1523/JNEUROSCI.16-21-06886.1996 PMC6579258

[40] Kirby BB, Takada N, Latimer AJ, Shin J, Carney TJ, Kelsh RN, et al In vivo time-lapse imaging shows dynamic oligodendrocyte progenitor behavior during zebrafish development. Nat Neurosci. 2006;9:1506–1511. doi: 10.1038/nn1803 17099706 10.1038/nn1803

[41] Gao FB, Durand B, Raff M. Oligodendrocyte precursor cells count time but not cell divisions before differentiation. Curr Biol. 1997;7:152–155. doi: 10.1016/S0960-9822(06)00060-1 9016704 10.1016/s0960-9822(06)00060-1

[42] Rosenberg SS, Kelland EE, Tokar E, De la Torre AR, Chan JR. The geometric and spatial constraints of the microenvironment induce oligodendrocyte differentiation. Proc Natl Acad Sci U S A. 2008;105:14662–14667. doi: 10.1073/pnas.0805640105 18787118 10.1073/pnas.0805640105 PMC2567234

[43] Kleinsimlinghaus K, Marx R, Serdar M, Bendix I, Dietzel ID. Strategies for repair of white matter: influence of osmolarity and microglia on proliferation and apoptosis of oligodendrocyte precursor cells in different basal culture media. Front Cell Neurosci. 2013;7:277 doi: 10.3389/fncel.2013.00277 24421756 10.3389/fncel.2013.00277 PMC3872727

[44] van Heyningen P, Calver AR, Richardson WD. Control of progenitor cell number by mitogen supply and demand. Cur Biol. 2001;11:232–241. doi: 10.1016/S0960-9822(01)00075-6 10.1016/s0960-9822(01)00075-6 11250151

[45] Sim FJ, Zhao C, Penderis J, Franklin RJ. IThe age-related decrease in CNS remyelination efficiency is attributable to an impairment of both oligodendrocyte progenitor recruitment and differentiation. J Neurosci. 2002;22:2451–2459. 11923409 10.1523/JNEUROSCI.22-07-02451.2002 PMC6758320

[46] Gautier HO, Evans KA, Volbracht K, James R, Sitnikov S, Lundgaard I, et al Neuronal activity regulates remyelination via glutamate signalling to oligodendrocyte progenitors. Nat Commun. 2015;6:8518 doi: 10.1038/ncomms9518 26439639 10.1038/ncomms9518 PMC4600759

[47] Hanahan D, Weinberg RA. Hallmarks of cancer: the next generation. Cell. 2011;144:646–674. doi: 10.1016/j.cell.2011.02.013 21376230 10.1016/j.cell.2011.02.013

[48] Enderling H, Hahnfeldt P. Cancer stem cells in solid tumors: is’evading apoptosis’ a hallmark of cancer? Prog Biophys Mol Biol. 2011;106:391–399. doi: 10.1016/j.pbiomolbio.2011.03.007 21473880 10.1016/j.pbiomolbio.2011.03.007

[49] Bogdańska MU, Bodnar M, Piotrowska MJ, Murek M, Schucht P, Beck J, et al A mathematical model describes the malignant transformation of low grade gliomas: Prognostic implications. PLoS One. 2017;12:e0179999 doi: 10.1371/journal.pone.0179999 28763450 10.1371/journal.pone.0179999 PMC5538650

[50] Murray JD. Mathematical biology. II: Spatial models and biomedical applications. 3rd ed Berlin: Springer-Verlag; 2002.

