# Supplementary material for: Modeling the dynamics of oligodendrocyte precursor cells and the genesis of gliomas
Source: PLoS Comput Biol. 2018 Mar 28;14(3):e1005977. doi: 10.1371/journal.pcbi.1005977 (PMC5903643; doi:10.1371/journal.pcbi.1005977)
Supplement: S1 Appendix — (PDF) [file pcbi.1005977.s001.pdf]

Modeling the dynamics of oligodendrocyte precursor cells and the genesis of gliomas: supplementary material

1. The simplified model

In order to study these oscillations, we introduced a simplified version of the model, aiming at keeping only the characteristic features that lead to oscillations in the model, but also in order to facilitate the derivation of the continuum limit. In this case, the three dimensional space is discretised into sites. Each cell occupies only one site (the cells are point-wise in this version). At each iteration, each cell can undergo either proliferation or differentiation/death (as explained in the text, we do not distinguish the two processes).

The proliferation rule is the same as in the full model. At each iteration, the following procedure is repeated for each cell: one of the 6 neighboring sites (in the 3D Von Neumann neighborhood) is chosen at random and a random number r is picked. If $r < \lambda$ and if the chosen neighboring site is free, the cell divides and a new cell is created in the previously chosen neighboring site. If the chosen site is already occupied by a cell, or if $r \geq \lambda$, the cell does not divide. Since cells are point-wise, overlaps are not possible anymore. Therefore, the density-dependent differentiation rule becomes: if the cell has one or more neighboring sites that are occupied, the lifetime clock is triggered.

When at least two out of the 6 neighbor sites of a cell are filled with a cell (i.e when a cell has more than two neighboring cells), the lifetime clock is triggered. After the triggering, the cell timer increases by a unit at each iteration of the cellular automaton. When the clock reaches the lifetime threshold (that is fixed at the beginning of the simulation and that is the same for all the cells) D, the cell differentiates/dies.

2. The continuous equations

Cellular automaton simulations, dealing with microscopic quantities like individual cells, are expected to be closer to reality. However they are computationally intensive, let alone for the fact that one must perform a considerable number of simulations in order to wash away the effect of fluctuations. It is thus intersting to seek directly equations governing the various macroscopic quantities. These equations are usually obtained through a coarse-graining procedure. If one considers the lattice as composed by subdomains over which the macroscopic quantities do not fluctuate much it is possible by proceeding to a continuum limit to obtain differential equations governing the spatial behaviour of said quantities. Similar considerations allow one to take a continuum limit for the time evolution.

The procedure just described, also known under the name of hydrodynamic limit, could in principle be applied to our cellular automaton model. However in the present case it is simpler to establish the equations of motion is a empirical way by following the same dynamics prescription as for the cellular automaton. The first hypothesis is that

the various macroscopic quantities do not have any spatial dependence. This is quite plausible since in the case of the cellular automation we do not consider cell migration and the only displacements are the ones immediately following cell fission. Thus the macroscopic quantities will depend only on time. We first introduce the mean cell density $w(t)$ (corresponding to the density of occupied sites in the cellular automaton). We assume that cells proliferate under the assumption of finite capacity and thus we represent this mechanism by a logistic law of the form $w' = \lambda w(1-w)$, where the prime represent the derivative with respect to time and the density has been normalised so as to have a capacity of 1. Next we assume that cell differentiation/death occurs only when a cell enters into contact with another one and that it occurs a certain (fixed) time after contact. Thus it is natural to distinguish those cells which have not have entered yet into any contact and for which a first contact starts the lifetime clock. We represent the density of the latter cells by $c(t)$ and represent the evolution of the system w, c by the equations

$$w' = \lambda w(1-w) - \tau \widetilde{w}\widetilde{c} \tag{1a}$$

$$c' = \lambda w(1-w) - \tau wc \tag{1b}$$

where the tilde indicates a delayed term i.e. while the non-tilded terms are taken at time t the ones with the tilde correspond to time $t-D$, i.e. $\widetilde{w} = w(t-D)$. Equations need some further explanation as far as the terms with negative sign are concerned. In equation (1b) the term $-wc$ represents the cells which enter into contact with others and start the process of **differentiation/death** which will occur after a time D. Thus the interaction term is taken at local time. The loss of cells through **differentiation/death**, modifying the total population, is the consequence of contacts that took place previously and thus the interaction term in (1a) is delayed by a quantity D. Moreover, by a suitable rescaling of time (and redefinition of λ and D) we can put the strength of the interaction term $\tau = 1$. Thus the system (1) has just two parameters: the proliferation parameter λ and the delay D. It is interesting to remark that by subtracting the two equations and integrating over time we obtain the conservation-like relation

$$c(t) - w(t) + \int_{t-D}^{t} w(s)c(s)ds = 0 \tag{2}$$

We proceed now to the study of the stability of the evolution equations. The system (1) possesses two fixed points corresponding to the possible stationary regimes. The obvious one is $w_\infty = 0$, $c_\infty = 0$ i.e. a totally empty space. In order to find the second one we assume that w_∞ is not zero in which case we obtain, from either (1a) or (1b), the relation

$$c_\infty = \lambda(1 - w_\infty) \tag{3}$$

and, using equation (2), we find for w_∞

$$Dw_\infty^2 - w_\infty(D - 1 - 1/\lambda) - 1 = 0 \tag{4}$$

which possesses only one positive real root. In order to proceed further we assume that the two parameters of the problem, λ and D, obey the relation $1 << 1/\lambda << D$. In

this case it is possible to obtain an approximate solution of (4) which, when we keep the first few terms, leads to the expressions

$$w_\infty = 1 - \frac{1}{\lambda D} + \frac{1}{\lambda D^2} \tag{5}$$

$$c_\infty = \frac{1}{D} - \frac{1}{\lambda D^2} \tag{6}$$

for the fixed point. It is straightforward to verify that, since $\lambda > 0$, the fixed point (0,0) is unstable. In order to study the stability of the non-trivial fixed point, given by (4) and the positive root of (5), we linearise (1a) and (1b) considering a small amplitude oscillation around the fixed point. We put $w = w_\infty + \xi$ and $c = c_\infty + \eta$ and obtain, using (4), the system

$$\xi' = \lambda(1 - 2w_\infty)\xi - \lambda(1 - w_\infty)\tilde{\xi} - w_\infty\tilde{\eta} \tag{7}$$

$$\eta' = -\lambda w_\infty \xi - w_\infty \eta \tag{8}$$

Next we look for a solution of the form $\xi = A\exp(\rho t)$, $\eta = B\exp(\rho t)$ and obtain the system

$$A\rho = \lambda(1 - 2w_\infty)A - \lambda(1 - w_\infty)Ae^{-\rho D} - w_\infty Be^{-\rho D} \tag{9}$$

$$B\rho = -\lambda w_\infty A - w_\infty B \tag{10}$$

which leads to the characteristic equation

$$\rho^2 + \rho(\lambda(1 - w_\infty)e^{-\rho D} - \lambda(1 - 2w_\infty) + w_\infty) + \lambda w_\infty(1 - 2w_\infty)(e^{-\rho D} - 1) = 0 \tag{11}$$

Next we introduce the dimensionless quantity $z = \rho D$ and rewrite (11) as

$$z^2 + zD(\lambda(1 - w_\infty)e^{-z} - \lambda(1 - 2w_\infty) + w_\infty) + \lambda D^2 w_\infty(1 - 2w_\infty)(e^{-z} - 1) = 0 \tag{12}$$

Equation (12) can be solved numerically once λ and D are given. However it is interesting within the assumptions we have introduced, namely, $1 << 1/\lambda << D$, to obtain an approximate solution for z, using the fact that $w_\infty \approx 1 - 1/(\lambda D)$. At lowest order (12) reduces to just

$$e^{-z} - 1 = 0 \tag{13}$$

the, lowest, non-trivial solution of which is $z = 2i\pi$. This means that at lowest order we have undamped oscillations with period $T = D$. Computing the next order in the small parameters we find

$$z = -\frac{2\pi^2}{\lambda^2 D^2} + 2i\pi\left(1 - \frac{1}{\lambda D}\right) \tag{14}$$

This means that we have now damped oscillations with period

$$T = \frac{D}{1 - 1/(\lambda D)} \tag{15}$$

We compared the period and the value of the density of occupied sites at equilibrium w_∞ when varying the lifetime clock threshold and the proliferation coefficient, between the cellular automaton with spheres, the simplified cellular automaton and the expression given by continuous model, equations (5) and (15), see S1 Figure.
