# Supplementary figures and images for: Modeling the dynamics of oligodendrocyte precursor cells and the genesis of gliomas
Source: PLoS Comput Biol. 2018 Mar 28;14(3):e1005977. doi: 10.1371/journal.pcbi.1005977 (PMC5903643; doi:10.1371/journal.pcbi.1005977)

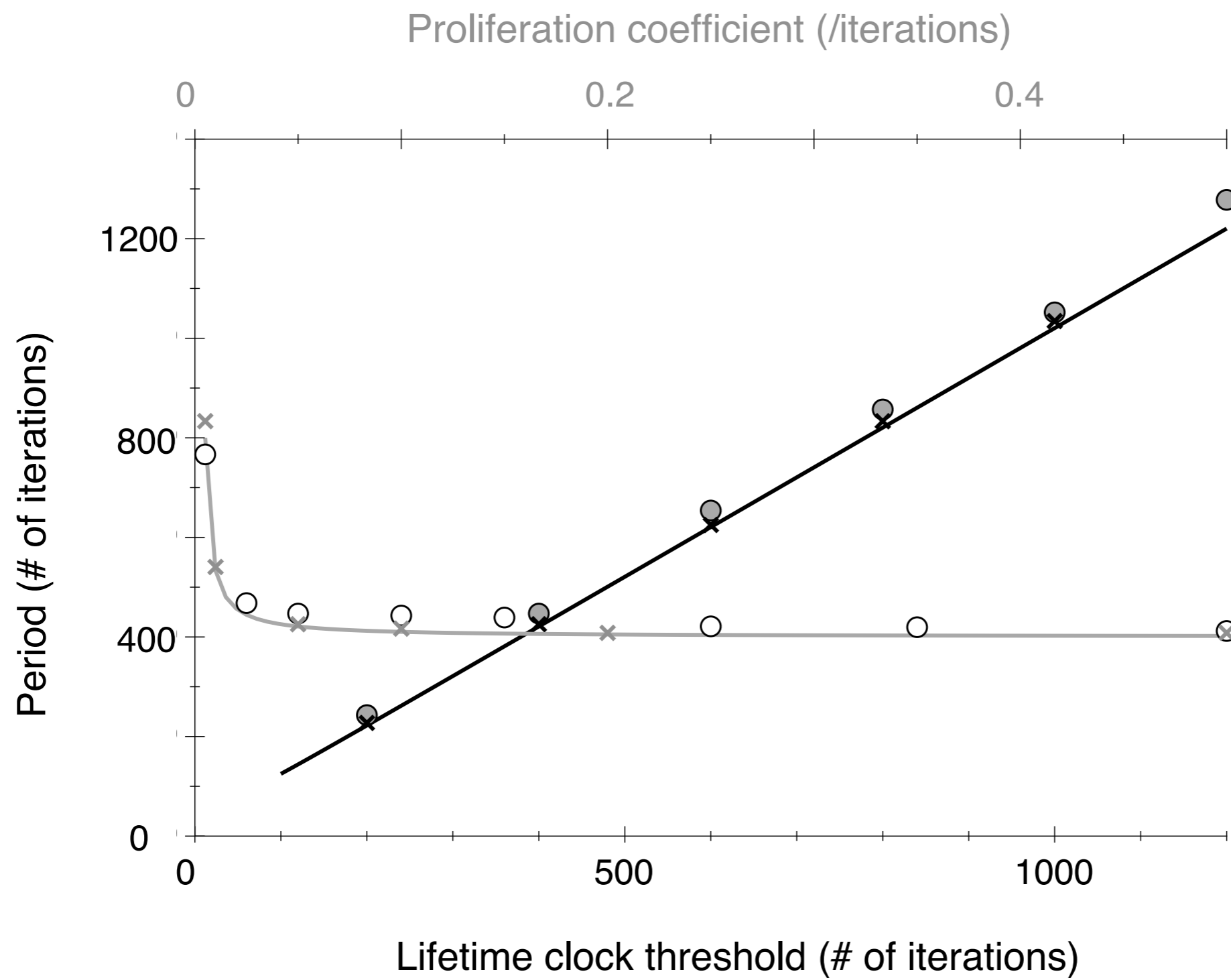

Supplement: S1 Fig — Period of oscillations versus the lifetime clock threshold (black crosses, line and full circles) and the proliferation coefficient (gray crosses, line and open circles), calculated from the analytical formula (5) (lines), from the simplified cellular automaton with point-wise cells (crosses) and from the cellular automaton with spheres (circles). (PDF) [file pcbi.1005977.s002.pdf]
